# Model-based stratification of progression along the Alzheimer disease continuum highlights the centrality of biomarker synergies

**DOI:** 10.1186/s13195-021-00941-1

**Published:** 2022-01-24

**Authors:** Muhammad Usman Sadiq, Kichang Kwak, Eran Dayan, for the Alzheimer’s Disease Neuroimaging Initiative

**Affiliations:** 1 Biomedical Research Imaging Center (BRIC), UNC-Chapel Hill, Chapel Hill, NC 27599 USA; 2 Department of Radiology, UNC-Chapel Hill, Chapel Hill, NC 27599 USA

**Keywords:** Alzheimer’s disease, AT(N) biomarkers, Deep learning, Cognitive phenotypes

## Abstract

**Background:**

The progression rates of Alzheimer’s disease (AD) are variable and dynamic, yet the mechanisms that contribute to heterogeneity in progression rates remain ill-understood. Particularly, the role of synergies in pathological processes reflected by biomarkers for amyloid-beta (‘A’), tau (‘T’), and neurodegeneration (‘N’) in progression along the AD continuum is not fully understood.

**Methods:**

Here, we used a combination of model and data-driven approaches to address this question. Working with a large dataset (*N* = 321 across the training and testing cohorts), we first applied unsupervised clustering on longitudinal cognitive assessments to divide individuals on the AD continuum into those showing fast vs. moderate decline. Next, we developed a deep learning model that differentiated fast vs. moderate decline using baseline AT(N) biomarkers.

**Results:**

Training the model with AT(N) biomarker combination revealed more prognostic utility than any individual biomarkers alone. We additionally found little overlap between the model-driven progression phenotypes and established atrophy-based AD subtypes. Our model showed that the combination of all AT(N) biomarkers had the most prognostic utility in predicting progression along the AD continuum. A comprehensive AT(N) model showed better predictive performance than biomarker pairs (A(N) and T(N)) and individual biomarkers (A, T, or N).

**Conclusions:**

This study combined data and model-driven methods to uncover the role of AT(N) biomarker synergies in the progression of cognitive decline along the AD continuum. The results suggest a synergistic relationship between AT(N) biomarkers in determining this progression, extending previous evidence of A-T synergistic mechanisms.

**Supplementary Information:**

The online version contains supplementary material available at 10.1186/s13195-021-00941-1.

## Background

Alzheimer’s disease (AD) is a progressive neurodegenerative disorder which gradually impairs memory, cognition, and other vital functions [1]. Individuals along the AD continuum exhibit markedly heterogeneous progression rates as the disease advances [2, 3]. Both linear and non-linear progression of cognitive decline has been documented in AD [4, 5], with distinct progression profiles found among individuals [2, 3]. Still, the mechanisms that underlie the heterogeneity in AD progression rates remain incompletely understood.

The neuropathological hallmarks of AD are centered around the presence of amyloid-beta (Aβ) plaques and neurofibrillary tangles of hyperphosphorylated tau, which are believed to precede structural neurodegenerative changes in the brain [6, 7]. Links between cognitive decline in AD and biomarker levels for Aβ [8], tau [9], and atrophy/neurodegeneration [10] have been reported in the literature. However, with little exception, studies have focused on individual biomarkers rather than examining their synergies and combined contribution to progressive cognitive decline along the AD continuum. An accurate characterization of the mechanisms leading to heterogeneity in progression rates would nevertheless benefit from considering biomarkers for Aβ (‘A’), tau (‘T’), and neurodegeneration (‘N’) together, consistent with the recently proposed AT(N) framework [11, 12]. Yet, combining AT(N) biomarkers in a single model is not trivial, given their complex, non-linear relationships with one another and/or their relationship with cognitive decline [13, 14]. A modeling approach based on deep learning arises as a natural solution to this problem, given its ability to model complex and non-linear mappings [15, 16]. Deep learning models have emerged as a powerful tool recently in relevant tasks, such as differentiating between individuals with dementia and controls [17, 18], and classifying stable vs. progressive mild cognitive impairment (MCI) [15, 19–21].

**Fig. 1:**
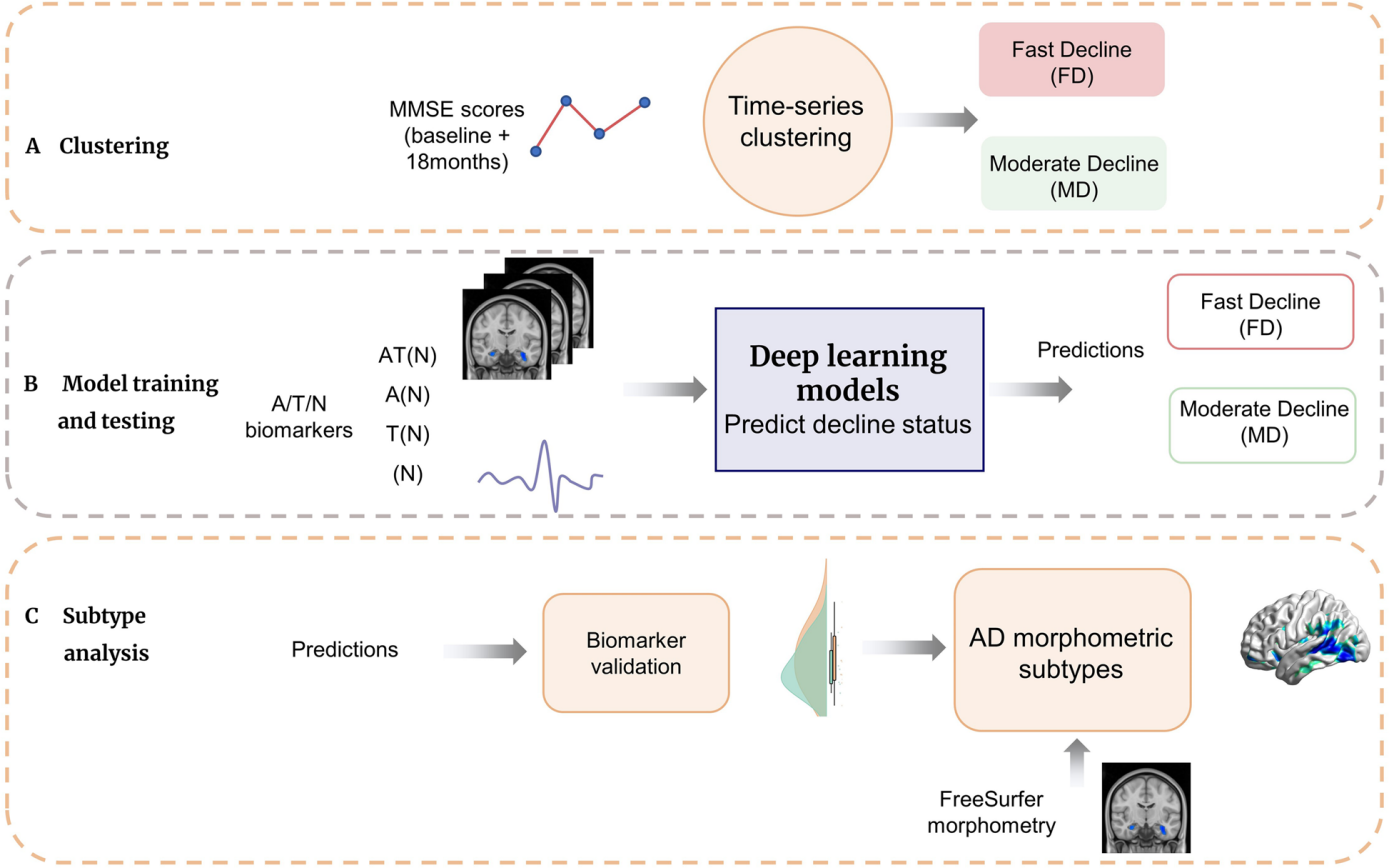
Study setup. **A** Clustering of MMSE scores to classify subjects as Fast and Moderate Decliners (FD and MD, respectively). **B** Baseline AT(N) biomarkers including CSF Aβ (A), CSF p-tau (T), and FDG-PET along with T1-weighted images (N), from a cohort of subjects with prodromal and clinical AD (*n *= 321, augmented to 1104) were used to train the deep learning models for FD/MD prediction. **C** The predicted cognitive progression phenotypes in the test set (*n*_test _= 97) were also examined for overlap with putative atrophy-based AD subtypes

In the current study, we propose a model-driven approach, based on AT(N) biomarkers, for stratifying progression rates along the AD continuum and delineating their underlying mechanisms. Notably, this work focuses on heterogeneity of cognitive decline along the AD continuum unlike previous studies where MCI progression was examined [22, 23]. We first employ data-driven clustering of cognitive assessments to define individuals with prodromal or clinical AD as either Fast Decliners (FD) or Moderate Decliners (MD) (Fig. 1A). These progression phenotypes are then used to train, validate, and test a deep learning model using baseline biomarkers for A (CSF Aβ _1–42_), T (CSF p-tau _181_), and N (MRI images and FDG-PET) (Fig. 1B). The model was trained with and without Aβ, tau, and neurodegeneration biomarkers, allowing us to compare the relative contribution of biomarker synergies, particularly amyloid-, and tau-mediated neurodegeneration to progression rates along the AD continuum. We additionally examined the extent to which the cognitive progression phenotypes predicted by our model reflected variation in regional atrophy characteristics (Fig. 1C), commonly used for subtyping AD [24–27]. This allowed us to examine if our model-based framework reflected patterns of neurodegeneration captured by other commonly used approaches.

## Methods

### time points during 24 months following baseline, (3) had T1-weighted MRI images takenParticipants and data acquisition

Data used in this study were obtained from the Alzheimer’s Disease Neuroimaging Initiative (ADNI) database (adni.loni.usc.edu; clinical trial registration: NCT00106899). The ADNI was launched in 2003 as a public-private partnership, led by the Principal Investigator Michael W. Weiner, M.D. The primary goal of the ADNI has been to test whether serial MRI, positron emission tomography (PET), other biological markers, and clinical and neuropsychological assessments can be combined to measure the progression of MCI and early AD. In the current study, participants from the ADNI-1, ADNI-2/Go, and ADNI-3 cohorts were included if they were (1) diagnosed with AD at baseline or within 1 year of their first diagnosis (i.e., MCI subjects were included if they were later diagnosed as AD), (2) had valid cognitive evaluations (Mini-Mental State Examination (MMSE) scores) performed at a minimum of *t* = 4 time points during 24 months following baseline, (3) had T1-weighted MRI images taken using 3T scanners based on either an inversion recovery-fast spoiled gradient recalled (IR-SPGR) or a magnetization-prepared rapid gradient-echo (MP-RAGE) sequences, and (4) were determined to be amyloid-positive within the study’s timeline according to published criteria (CSF Aβ < 976.6 pg/mL or ^18^F-florbetapir-PET uptake ratio > 1.11). The ^18^F-florbetapir-PET uptake ratios, provided in ADNI as a derived variable, were calculated by extracting weighted cortical retention means from frontal, cingulate, parietal, and temporal regions, after co-registering the PET and MRI scans. These data were used to calculate standardized uptake value ratios (SUVRs), normalized by a whole cerebellum reference region. SUVRs with a positivity threshold of 1.11 were then identified [28, 29].

In total, 321 unique subjects were identified using these criteria. Out of these, 310 subjects were determined to be amyloid-positive using CSF Aβ cutoff while 11 subjects satisfied the PET uptake ratio criterion. All subjects provided written informed consent, and the procedures were approved by the Institutional Review Boards of participating centers.

### MRI images and their processing

T1-weighted SPGR or MPRAGE images were acquired using 3T scanners (full details of the image acquisition protocols can be found online (http://adni.loni.usc.edu/methods/documents/mri-protocols/). T1-weighted images were used for training of the deep learning models. The cohort with valid MRI and cognitive assessments were split between the training (*n*_train_ = 224) and testing (*n*_test_ = 97) datasets.

CSF Aβ _1-42_ (amyloid-beta) and p-tau_181_ (tau) biomarkers along with MRI + fluorodeoxyglucose (FDG)-PET data (neurodegeneration) were used as the A, T, and N biomarkers respectively in our AT(N)-centered analytical framework. CSF samples used in this study were collected and processed previously (see, [30]; http://adni.loni.usc.edu/methods). CSF Aβ and p-tau were measured with the fully automated Elecsys immunoassay (Roche Diagnostics, Basel, Switzerland) by the ADNI biomarker core (University of Pennsylvania, Philadelphia, PA). Processed (see, http://adni.loni.usc.edu/methods) FDG PET images were averaged, with uptake values from angular, temporal, and posterior cingulate cortices serving as one of our two biomarkers for neurodegeneration (along with MRI) [31]. This average FDG PET was previously obtained using a series of steps to mitigate inter-scanner variability and normalized in spatial resolution and intensity range for further analysis [32]. Each MRI image was standardized to 0 mean and unit standard deviation. Similarly, other AT(N) biomarkers were standardized before being used as input in the deep learning model.

### Unsupervised clustering of cognitive measurements

To characterize longitudinal change in cognition, we used 2-year follow-up MMSE scores. In addition, other cognitive assessments over the same duration were used for validation purposes, including the Alzheimer’s Disease Assessment Scale, 13-Item Subscale (ADASCog13), Clinical Dementia Rating Sum of Boxes (CDR-SB), and Functional Assessment Questionnaire (FAQ). These tests were administered as described online (http://www.adni-info.org).

We used time-series clustering based on the dynamic time-warping (DTW) method [33] to identify cognitive phenotypes in a data-driven manner. Clustering is typically applied in order to partition a heterogeneous set of samples into more homogeneous clusters based on some similarity measure. When it comes to clustering of time-series data, a DTW-based similarity measure is more widely applicable than the conventional Euclidean distance or spatial distance based measures [34, 35]. DTW is able to find optimal global alignment between sequences of different shapes. The shape-based DTW method is particularly well-suited to dynamic time-series data with potential temporal drift, showing better accuracy than linear models [34, 36]. We used the DTW to cluster the MMSE scores of our cohort using *t* = 4 time points, collected over 2 years from baseline, using Hierarchical Agglomerative Clustering with Ward’s linkage [37]. Clustering was repeated with other linkage methods such as Ward1 and the unweighted pair-group method using arithmetic averages (UPGMA) to examine the similarity of cluster labels [38, 39]. Other cognitive assessments such as ADASCog13, CDR-SB, and FAQ were used for validation purposes, testing if the phenotypes based on MMSE scores also differ in other measures of cognition in AD. Further, to determine the optimal number of cognitive decline clusters in our cohort, we used silhouette analysis to compare average silhouette width for *k* = 2, 3, and 4 clusters.

### Deep learning model architecture and training

Deep learning models have been extensively used for AD classification [17, 18] and predicting progression of MCI [15, 21, 40]. Deep learning models are typically compared against linear or non-linear Support-Vector Machine (SVM), logistic regression, or random forest classifiers, where SVM has been shown to outperform the latter two [41]. We first calibrated our deep learning model’s performance using a similar comparison with SVM. Our deep learning model used a parameter-efficient architecture similar to that previously proposed for classification of MCI [42]. The Parameter-Efficient Network model, designated as PENet, takes a combination of baseline AT(N) biomarkers including MRI images and FDG-PET (*N*), CSF p-tau (*T*), and CSF Aβ (*A*) and learns to predict the subject’s cognitive decline status (FD vs MD) using these baseline measurements only. The multi-modal feature extractor implemented in the model uses a series of convolutional blocks, or conv blocks, to process MRI tensors. These conv blocks are composed of a convolutional layer followed by batch normalization and exponential linear unit (ELU) transformation. The model also makes use of separable convolution blocks, or sep-conv blocks, which perform the operation of a convolution block but with far fewer parameters, hence reducing the risk of over-fitting. PENet uses 2 conv blocks followed by 3 sep-conv blocks with increasing number of filters (Fig. 3A). It processes non-imaging biomarkers by dense or FC (fully connected) blocks.

#### Implementation

Experiments were conducted using python version 3.6. The implementation was developed using the Keras deep learning library with Tensorflow backend. The model was trained on Ubuntu 18.04 on a single Nvidia Tesla V100 GPU with 16G memory, using a batch size of 25 and trained for 50 epochs after which the model showed stable dynamics (Fig. S2). This training was performed using the Stochastic Gradient Descent algorithm with an initial learning rate = 8 × 10^-4^ and exponential decay with a drop rate = 0.5. The FC layers used in the model were regularized using *L*_2_ regularization with penalty coefficient = 5 × 10^-4^.

#### Data augmentation and validation framework

The implemented model was trained and validated using 5-fold cross-validation stratified by class phenotypes. All qualifying subjects from ADNI-1, ADNI-2/Go, and ADNI-3 were used in our experiments, yielding a total of *n* = 321 subjects (*n*_train_ = 224, MD = 136, FD = 88; *n*_test_ = 97, MD = 58, FD = 39). To improve model generalizability, we augmented the training dataset through a combination of image rotation (random angle in [−90°, 90°], translation (random shift in [0, 0.5]), and flipping operations, resulting in 1104 training images. Special care was taken to use the test dataset only after all steps of augmentation, model selection, and hyperparameter tuning were completed, ensuring no data leakage.

### Analysis of atrophy-based AD subtypes

MRI images for the test dataset were processed (http://adni.loni.usc.edu/) using Freesurfer (http://surfer.nmr.mgh.harvard.edu/) to extract region of interest (ROI)-based gray matter (GM) volume. The following processing steps were performed: (1) motion-correction and skull-stripping based on a watershed deformation method [43], (2) image registration to the Talairach brain template, (3) estimation and labeling of gray matter-white matter (GM-WM) boundary using a tessellation step, and (4) registration of volume to an atlas to acquire volume and surface statistics for each ROI. Using these extracted volumes, we investigated the potential association between the model-based cognitive progression phenotypes and atrophy-based AD subtypes, as previously identified [24]. Subtypes were identified using the ratio of hippocampal volume (HV) to cortical total volume (CTV). Following the same procedure as described previously [24], subjects in the test dataset with an HV:CTV ratio above the 75th percentile were identified as belonging to the Hippocampal-Sparing AD (HpSp) subtype, those with HV:CTV ratio below the 25th percentile as belonging to the Limbic-Predominant subtype (LP) and the rest were designated as typical-AD (tAD).

To visualize the spatial extent of atrophy, the subtypes HpSp, LP, and tAD were also contrasted against age-matched controls (*n* = 30) to extract voxel-wise contrast maps using FSL's optimized voxel-based morphometry (VBM) (http://www.fmrib.ox.ac.uk/fsl/) [44]. The FSL VBM processing pipeline involved brain extraction of T1-weighted images followed by segmentation into WM, GM, and CSF volume probability maps. Next, a random subset of each compared cohort was used to create the average study-specific GM template by registration to MNI152 space using the FSL FLIRT tool. This was followed by non-linear registration of all GM images in the native image space to the average GM template. Subsequently, these registered images were smoothed using a full-width half-maximum (FWHM) of 6mm and their voxel-wise GM volumes were contrasted using a general linear model (GLM) formulation. To identify significant differences between the compared groups, non-parametric statistics were performed using the ‘randomise’ FSL function (5000 permutations) with FWE correction set at *p* < 0.05, based on threshold-free cluster enhancement (TFCE).

### Statistical analysis

For comparisons between two groups, unpaired two-sided *t* tests or Wilcoxon rank-sum test were used. For testing significant differences in MMSE scores of the MD and FD phenotypes, we used the selective inference method [45] implemented in the R ‘clusterpval’ package, which controls for type I error rate in group comparisons after clustering. To analyze longitudinal changes in ADASCog13, CDR-SB, and FAQ scores, two-way repeated measures ANOVA was used to examine the main effect of time and its interaction with the MD/FD phenotypes.

## Results

### Clustering cognitive assessments according to progression rates

**Fig. 2:**
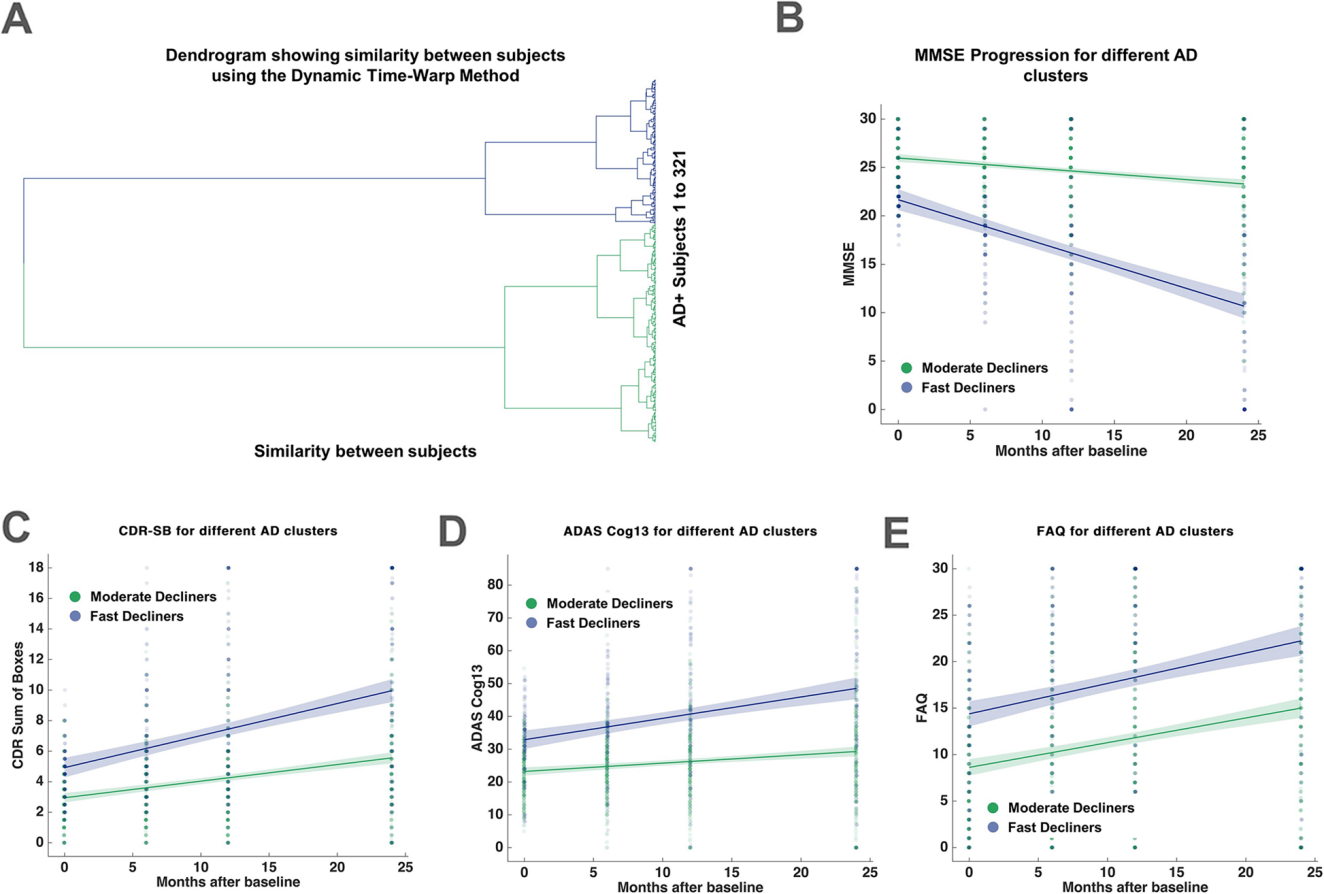
Time-series clustering of MMSE scores reveals varying rates of progression in subjects along the AD continuum. **A** Dendrogram showing the degree of similarity in MMSE time-series taken from individual subjects. **B** Longitudinal MMSE scores in individuals along the AD continuum over 24 months from baseline clustered into 2 distinct groups: Moderate and Fast Decliners (MD and FD) along with regression lines and 95% confidence intervals. **C** Variation of CDR-Sum of Boxes scores in the 2 progression phenotypes over 24 months from baseline. **D** Variation of ADAS Cog13 scores in the same subjects over 24 months from baseline. **E** Variation of FAQ scores in the same subjects over 24 months from baseline

Data used in this study were obtained from the ADNI database [46], combining participants from the ADNI-1, ADNI-2/GO, and ADNI-3 cohorts (*N* = 321; *m* = 180, *f* =141). We first applied data-driven clustering to derive distinct cognitive progression phenotypes from the sample, considering longitudinal (over 2-year follow-ups) changes in MMSE scores [47]. We used an unsupervised time-series clustering technique based on DTW with Ward’s linkage-based agglomerative clustering [33, 37], suitable for shape-based clustering of dynamic time-varying observations [34, 35]. The longitudinal MMSE scores of the entire cohort were clustered to reveal 2 different progression phenotypes identified as moderate (MD: *n* = 194; ages 73.8 ± 7.28; Supplementary Table 1) and fast (FD: *n* = 127; ages 73.2 ± 8.02) decline (Fig. 2A and B). Other linkage methods such as Ward1 [38] and UPGMA resulted in very similar clustering solutions [38, 39]. The 2 clusters did not exhibit any significant differences in age, education, gender, total cortical volume, and *APOE e4* status (all *p* > 0.05; Supplementary Table 1 and Fig. S3). Silhouette analysis, used to determine the optimal number of clusters, resulted in maximal silhouette width for *k* = 2 clusters (Supplementary Table 2). The MD and FD phenotypes showed, as expected, significant differences in MMSE profiles (*p* = 6.73 × 10^-3^), revealed using a method developed for post-clustering comparisons [45]. Subjects in the MD and FD phenotypes were also compared for longitudinal changes in ADASCog13, CDR-SB, and FAQ scores. Similar to the MMSE scores, the ADASCog13, CDR-SB, and FAQ scores showed distinct patterns of decline in the different progression phenotypes (Fig. 2C-E). Repeated measures ANOVA for ADASCog13 revealed a significant main effect of time (*F*(3, 960) = 48.14; *p* < 2 × 10^-10^) and a significant interaction between time and phenotype (*F*(3, 960) = 20.68; *p* < 2 × 10^-10^). Similarly, significant main effects for time as well as significant time by phenotype interactions were observed for CDR-SB (main effect: *F*(3, 960) = 85.55; *p* < 2 × 10^-12^; interaction: *F*(3, 960) = 24.46; *p* < 2 × 10^-12^), and FAQ scores (main effect: *F*(3, 960) = 75.49; *p* < 2 × 10^-13^; interaction: *F*(3, 960) = 3.13; *p* = 0.024).

### Deep learning model predictions and role of AT(N) biomarkers in progression along AD continuum

**Fig. 3:**
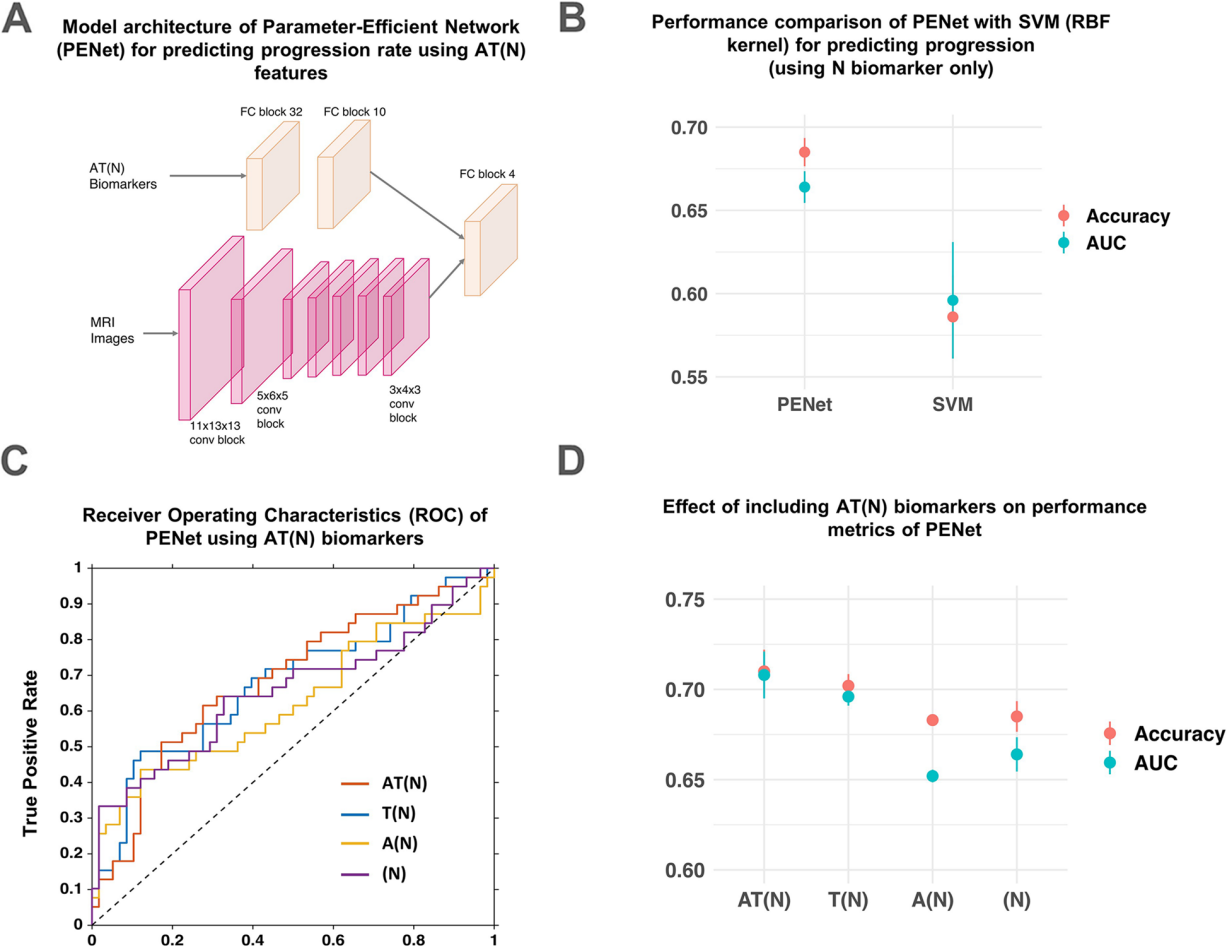
Parameter Efficient Net (PENet) for progression phenotype prediction and its performance. **A** Model architecture and layers of the PENet used here for predicting the rate of decline observed in individuals along the AD continuum (MD vs. FD). **B** Performance comparison of the PENet model with SVM (with Radial Basis Function kernel) for predicting progression along the AD continuum using (N) biomarkers shows PENet’s superiority. **C** Receiver operating characteristics (ROC) curve showing the performance of PENet using different combinations of A, T, and N biomarkers. **D** Performance metrics for the PENet show the effect of using different combinations of AT(N) biomarkers on prediction accuracy. The model’s performance progressively improved as A and T biomarkers were added to N; however, the addition of the A biomarker had no effect relative to a model based on N biomarkers alone

The FD and MD phenotypes identified by clustering were next used for model training, where a combination of baseline AT(N) biomarkers including MRI images and FDG-PET (*N*), CSF p-tau _181_ (*T*), and CSF Aβ _1–42_ (*A*) were used as input to the model. We constructed a parameter-efficient deep learning architecture similar to that previously proposed [42]. This architecture uses fewer parameters than most other models used in similar tasks with state-of-the-art performance and is well-suited for data-limited applications. The overall architecture makes use of 2 convolutional blocks followed by 3 separable convolution blocks with progressively increasing number of filters (Fig. 3A; See the “Methods” section for more details). In the model, non-imaging biomarkers are processed by dense or FC (fully connected) blocks. Finally, extracted features from MRI and biomarkers are combined through FC layers to generate predictions. The implemented model was trained and validated using 5-fold cross-validation stratified by cognitive phenotypes. All subjects meeting our criteria for analysis from ADNI-1, ADNI-2/Go, and ADNI-3 were used in the experiments, yielding a total of *n* = 321 subjects (*n*_train_ = 224, MD = 136, FD = 88; *n*_test_ = 97, MD = 58, FD = 39).

We first evaluated the performance of a basic PENet model trained using N features alone. The model showed significantly better performance than a random classifier when compared using DeLong’s test (*p* = 0.014; Fig. S1) [48]. The PENet model was then also compared with an SVM classifier, which used a radial-basis function kernel and N features as input. The PENet outperformed the SVM model (Fig. 3B), achieving an accuracy of 0.685 (area under the curve, AUC = 0.66).

Next, different combinations of AT(N) biomarkers were used in the PENet model to test their prognostic value and evaluate the role of biomarker synergies in progression along the AD continuum. This method of assessing the predictive successes of different biomarker combinations was preferred over linear or non-linear regression-based methods because of the possibility of complex, higher order synergies between AT(N) biomarkers, especially when 3D voxel-level data is included [14, 49]. Including all AT(N) biomarkers in the model yielded the best classification accuracy (0.710 ± 0.024), followed by T(N) (0.702 ± 0.013), N (0.685 ± 0.017), and A(N) (0.683 ± 0.006) (Fig. 3C, D). Individual A and T biomarkers, when used to predict progression phenotype using a logistic regression model, yielded only 59.7% and 56.7% classification accuracies (Supplementary Table 4). Thus, a comprehensive model based on A, T, and N was more accurate at predicting AD progression phenotypes than biomarker pairs (A(N) and T(N)) or individual biomarkers (A, T, or N). Moreover, relative to a model based on N features alone, the addition of A features resulted in slightly worse accuracy, while the addition of T features resulted in 1.7% improvement. In a secondary analysis, we also investigated the effect of including a proxy for brain reserve and excluding FDG-PET from the model’s inputs. In the first experiment, intracranial volume (ICV), a common proxy for brain reserve [50], was used as input to the model together with N biomarkers (MRI and FDG-PET). In the second experiment, FDG-PET was removed from the N biomarkers so that only MRI images were used as N inputs. The two experiments resulted in classification accuracies of 0.686 (± 0.021) and 0.677 (± 0.010) respectively, compared to that observed when using N biomarkers without these changes (0.685 ± 0.017) (Supplementary Table 5).

**Fig. 4:**
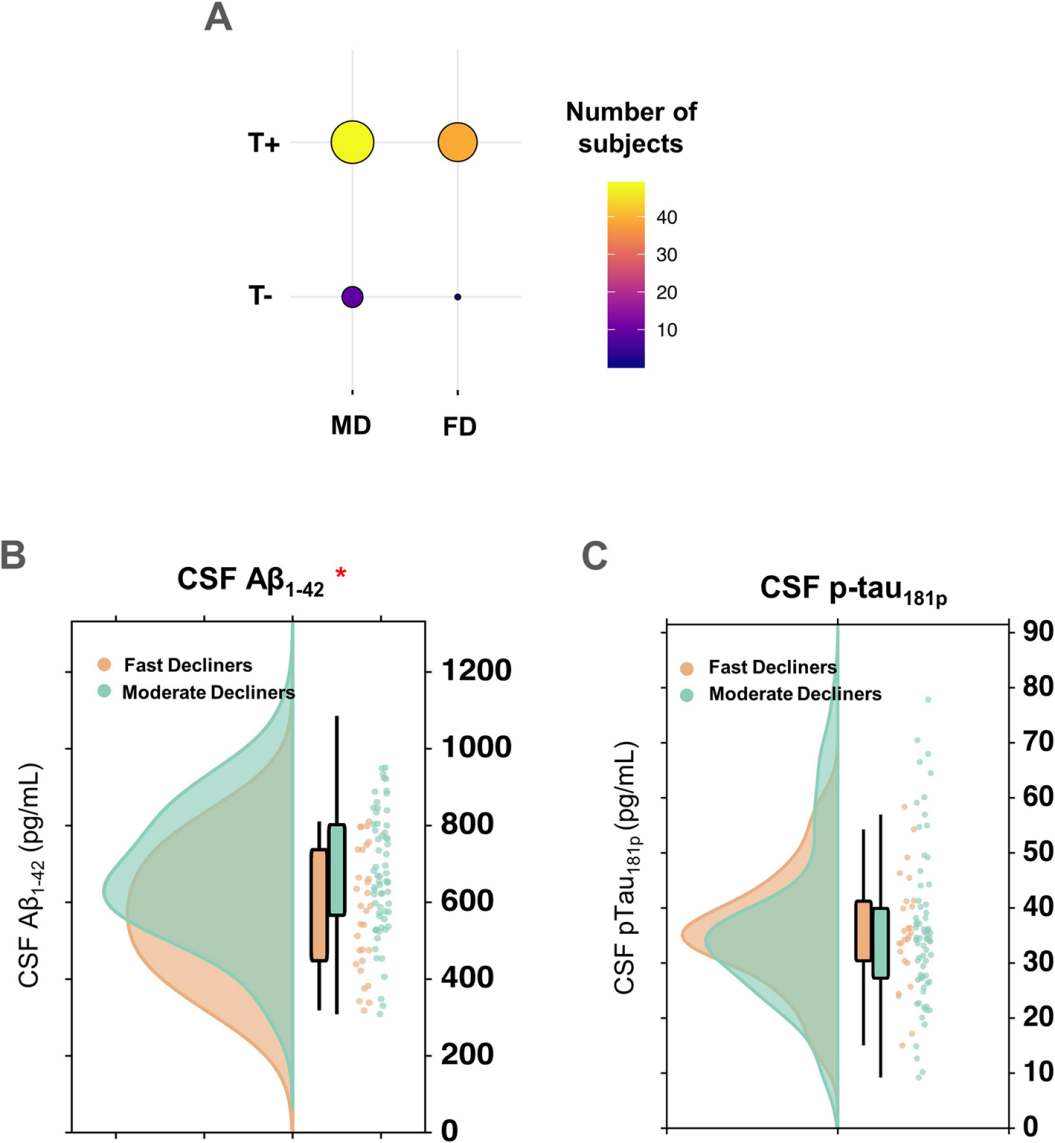
Differences in the role of CSF Aβ and CSF p-tau in progression along the AD continuum. **A** Contingency table showing significant difference in the ratio of tau positivity between the two progression phenotypes. **B** CSF Aβ differences in cognitive progression phenotypes predicted by a PENet model using T(N) biomarkers only. **C** CSF p-tau differences in cognitive progression phenotypes predicted by the PENet model using A(N) biomarkers only. *Significant differences (*p* < 0.05)

We next tested whether the predicted cognitive progression phenotypes differed in tau positivity (where T+ was defined as CSF p-tau > 21.8 pg/ml, [12]). We found significant differences in the proportion of tau positivity between the MD and FD groups (*χ*^2^= 4.48, *p* = 0.034; Fig. 4A). The differences between the predictive value of CSF p-tau and CSF Aβ were then further validated, by testing the degree of redundancy in their predictive value. To do this, the MD and FD phenotypes as predicted by A(N) biomarkers were tested for significant differences in CSF p-tau. Similarly, the two phenotypes as predicted by T(N) biomarkers were tested for significant differences in CSF Aβ. Significant differences were only found between CSF Aβ levels in the model where the MD/FD phenotypes were predicted using T(N) biomarkers (*p* = 0.02). No significant differences were observed between CSF p-tau levels in the model where the predicted MD/FD labels were based on A(N) biomarkers (Fig. 4B, C). Thus, the combination of T(N) features could account for the variance in CSF Aβ between the two phenotypes whereas the combination of A(N) features could not account similarly for the variation in CSF p-tau.

The moderate and fast decliners were distributed according to clinical diagnosis as follows: MD (MCI = 123, AD = 71) and FD (MCI = 22, AD = 105). Thus, there were more MCI subjects in the MD group, and more AD subjects in the FD group, respectively. Therefore, to examine if the model was only learning MCI/AD characteristics instead of reflecting the heterogeneity of cognitive decline along the AD continuum, we tested our model trained on MD/FD classification in the task of detecting the MCI/AD clinical diagnosis. Using the 5-fold cross-validation setup used in previous experiments, the model predicted the MCI/AD diagnosis with 0.584 ± 0.03 accuracy, showing substantially worse performance than that observed in the task of MD/FD classification. This demonstrates the distinct nature of the MD/FD and MCI/AD classification problems.

### Association of cognitive progression phenotypes with AD subtypes

**Fig. 5:**
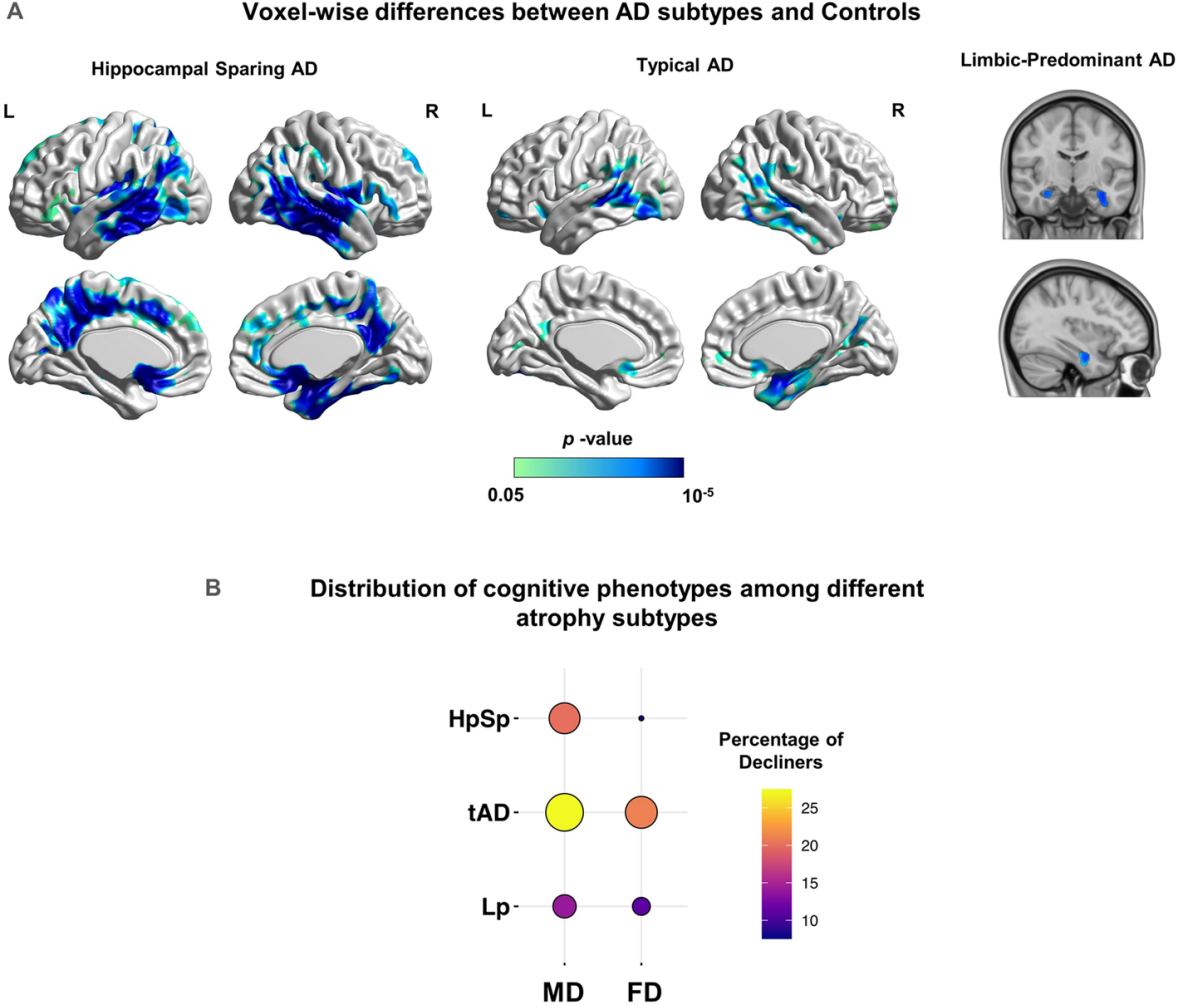
Association between progression phenotypes predicted by the deep learning model and AD subtypes identified by morphometric analysis. **A** Voxel-wise differences between the different AD subtypes and a group of age-matched healthy controls (*n*=30) reveal the regional atrophy patterns in these subtypes. The HpSp subtype (associated mostly with the FD phenotype) was manifested in widespread atrophy spread to the bilateral temporoparietal cortex, precuneus, and posterior cingulate regions while the LP subtype (corresponding mostly with the MD phenotype) displayed voxel-wise differences only in limbic regions. Voxel-wise differences were calculated using FSL-VBM with FWE correction set at *p* < 0.05, based on the threshold-free cluster enhancement (TFCE) statistic. **B** Contingency table showing the association between AD subtypes and the progression phenotypes predicted by the deep learning model. Overall, the different subtypes did not exhibit a significantly different distribution between the two progression phenotypes (*χ*^2^ test, *p* = 0.497)

Using extracted GM volumes for subjects in the test set (see the “Methods” section), we next investigated the potential association between the progression phenotypes identified here and atrophy-based AD subtypes as previously identified [24]. The objective of this analysis was to examine if the cognitive progression phenotypes predicted by our model reflected AD subtypes captured solely by patterns of neurodegeneration. The atrophy-based AD subtypes were defined based on the HV:CTV ratio. This resulted in a total of 26, 24, and 47 subjects assigned to the HpSp, LP, and tAD subtypes, respectively. First, to validate the presence of previously identified atrophy patterns in these subtypes, the spatial extent of their regional atrophy was visualized by contrasting them against data from age-matched controls (*n* = 30; Fig. 5A). These maps were obtained using the FSL VBM approach [44]. Consistent with previously observed atrophy patterns [51], the HpSp subtype was manifested in atrophy spread to bilateral temporoparietal cortex, precuneus, and posterior cingulate regions. On the other hand, the LP subtype displayed voxel-wise differences only in limbic regions. Next, we determined if the atrophy-based AD subtypes overlapped with the predicted cognitive progression phenotypes (MD/FD) identified via our modeling approach. We found no significant differences in the distribution of the different atrophy subtypes among the two cognitive progression phenotypes (*χ*^2^= 1.39, *p* = 0.497; Fig. 5B). Altogether, the HpSp subtype was more prevalent in the MD compared to the FD phenotype (19/7), while the tAD (26/21) and LP (13/11) subtypes were more equally distributed among the MD and FD phenotypes.

## Discussion

The mechanisms that underlie the marked heterogeneity in AD progression are to date incompletely understood. In the present work, we used a combination of data and model-driven methods to investigate heterogeneity along the AD continuum and tease apart the contribution of AT(N) biomarkers and their synergies to this progression. We first demonstrated that progression along the AD continuum varied considerably between individuals by applying unsupervised clustering on longitudinal cognitive assessments. Next, we used a parameter-efficient deep neural network (PENet) to predict the different cognitive progression phenotypes using baseline AT(N) biomarkers. Our model showed that the combination of all AT(N) biomarkers had the most prognostic utility in predicting progression along the AD continuum. A comprehensive AT(N) model showed better predictive performance than biomarker pairs (A(N) and T(N)) and individual biomarkers (A, T, or N). Finally, we report that the cognitive progression phenotypes did not overlap with previously established AD subtypes, defined solely based on patterns of neurodegeneration (atrophy). Altogether, our findings highlight a central role for concurrent AT(N) mechanisms which determine the rate of cognitive decline along the AD continuum.

Comparison of age, gender, education, total cortical volume, and *APOE e4* status at baseline revealed no significant differences between the various cognitive phenotypes identified using DTW clustering. This is noteworthy because of the concern that unsupervised clustering may capture variance not necessarily relevant to disease expression patterns, but rather may reflect confounds such as age, gender, or education. While baseline differences in MMSE scores between the phenotypes may have arisen due to differences in disease duration which can only be estimated indirectly in neurodegenerative disease [52], the accentuation of these differences with time indicates that the phenotypes declined at a significantly different rate. Our findings of discrete cognitive progression phenotypes are consistent with previous work on identifying distinct cognitive subtypes in AD [3, 53]. However, our clustering approach is specifically suitable for finding optimal global alignment between time-series data. Since cognitive phenotypes have been suggested to progress at different speeds in various stages of the disease (linearly or non-linearly) [4, 5], more complex modeling methods such as DTW are needed to capture the shape of the progression curve, taking into account potential temporal shifts.

The cognitive phenotypes identified by our analysis also differed significantly in ADASCog13, CDR-SB, and FAQ scores at the 2 years mark. Consistent with our results, differences in ADASCog13, CDR-SB, and FAQ scores were found among some or all of the cognitive subtypes identified in previous studies [24, 54, 55]. In other studies where a more detailed profiling of memory and cognitive domains was included, atypical subtypes were also identified based on visuospatial functioning and language impairment [56, 57]. Moreover, our finding of two distinct cognitive phenotypes is supported by previous studies [3, 58], which have robustly identified two cognitive clusters across multiple AD dementia cohorts, using a data-driven approach.

The MD and FD phenotypes identified via clustering were treated as labels in a model-driven classification and prediction of subjects’ cognitive progression phenotype using baseline N biomarkers. Comparison of the deep learning model with an SVM classifier showed better performance for the former model, similar to results published in similar tasks such as MCI classification [41, 59]. Using the PENet deep learning model and neurodegeneration biomarkers only (T1-weighted images and FDG-PET) resulted in baseline accuracy of 0.685, which progressively increased to 0.710 when adding additional T/N biomarkers. Critically, a comprehensive/complete AT(N) model showed more predictive power than biomarker pairs (A(N) and T(N)) and individual biomarkers (A, T, or N), suggesting that complex synergies between all three biomarker types underlie the progressions of cognitive decline along the AD continuum. This finding extends the emerging evidence from animal model and human studies on synergies between Aβ and tau [60, 61]. For example, injection of Aβ_42_ fibrils into the brains of P301L mutant tau transgenic mice caused a substantial increase in the numbers of neurofibrillary tangles both near the injection site and in regions projecting to it [62]. Evidence pointing to synergies between Aβ and tau also comes from studies in humans [63, 64]. Tau spread outside entorhinal cortex is enhanced by Aβ deposition in cognitively normal older adults [65]. Moreover, significant interactions between CSF Aβ and CSF p-tau affecting brain structure were reported in preclinical AD [66]. Similarly, findings based on PET imaging demonstrate that both Aβ and tau underlie memory decline in preclinical AD [64]. Altogether, previous evidence on synergies between Aβ and tau have mostly originated from studies in cognitively normal or preclinical AD populations [64, 67]. Our study extends this work,  examining progression along the AD continuum. Moreover, our results suggest that synergies between all three biomarker types (Aβ, tau, and neurodegeneration) underlie the progression of cognitive decline along the AD continuum. Our deep learning model and its reliance on AT(N) features is also distinct from previous machine learning models where multi-modal AD classification was based on N features (e.g., MRI and FDG) [40, 68] or combined cognitive or demographic variables [42, 69]. Further, previous machine learning based models have been mostly deployed to distinguish AD/CN or predict progression of MCI (see [15, 41] for a review), whereas our investigation focused on cognitive progression phenotypes in the AD continuum.

Our results reveal that the addition of A (Aβ) biomarkers to the model resulted in effectively no increase in accuracy, while the inclusion of T (tau) biomarkers resulted in an improvement of 1.7% in accuracy. This role of T biomarkers in progression along the AD continuum was further highlighted by the significant differences in tau positivity found between the MD and FD phenotypes. Thus, evidence suggests that CSF p-tau is a stronger determinant of progression rate along AD continuum than CSF Aβ, consistent with previous reports showing that tau-mediated neurodegeneration mechanisms result in heterogeneous AD progression [9, 70]. This observation of tau-associated progression along the AD continuum is also in agreement with previous studies where MCI progression was linked to elevated CSF p-tau [71, 72]. Further, the addition of ICV as an N biomarker in the model did not result in a meaningful change in classification accuracy (+0.1%), likely due to the covariance between ICV and voxel-level N biomarkers. Similarly, the exclusion of FDG-PET from the model’s N biomarkers had a marginal effect on accuracy (−0.8%).

Since the inclusion of more variables did not always improve the prediction accuracy of the model, the performance differences between the model based on AT(N) inputs and those obtained when using individual inputs cannot be explained solely by the number of input variables in the model. Additionally, the question of whether Aβ, tau, and neurodegeneration biomarkers have an additive or interactive relationship can improve our understanding of the disease. Our results indicate that performance gains associated with the addition of the A biomarker to a model trained on T(N) inputs is different from the improvement gained by adding A to (N) inputs only. In other words, performance improvement due to A is a function of whether T is included in the model’s inputs, which suggests that the A and T synergies found in our model are non-additive in nature. We, however, acknowledge that more formal ways of testing interaction vs. additive effects such as partial dependency plots should be employed in future studies to investigate the nature of biomarker synergies in progression along the AD continuum.

The deep learning model deployed here demonstrated good discriminative performance, where special care was taken to avoid different sources of data leakage previously identified [41]. Deep learning models have been extensively used for predicting progression of MCI to AD [15, 19, 21], along with other related tasks such as AD classification with missing data [73] and early detection of AD [74]. However, little is known about their utility in identifying cognitive progression phenotypes using the AT(N) framework. Employing this approach allowed us to investigate the complex and potentially non-linear relationships between these biomarkers. Furthermore, the shape-based DTW clustering of cognitive assessments, as applied here, allowed us to more optimally capture progression rates in AD, as it is specifically designed for modeling time-varying data [36].

We tested whether the cognitive progression phenotypes were captured by an established subtyping approach [24] based solely of patterns of neurodegeneration. We found no significant differences in the distribution of AD subtypes among the two cognitive progression phenotypes. Thus, the model-based phenotypes identified here, may not be readily detectable using atrophy-based methods. Data-driven studies on AD subtyping revealed neurodegeneration patterns similar to those found here [24–26], but atrophy-based methods do not always result in distinct cognitive progression phenotypes [27]. Additional work is needed to better reconcile atrophy and cognitive-based subtyping of AD and its progression.

## Limitations

Several limitations should be noted. First, we acknowledge that any study investigating the effects of AT(N) biomarkers in AD should ideally test longitudinal changes in these biomarkers in the same cohort. However, this was not applicable in the current study due to missing data in several of the biomarkers. Second, while being beyond the scope of the current study, an examination of the spatiotemporal characteristics of amyloid and tau deposition using PET-based markers can provide useful information about the progression of cognitive decline along the AD continuum. Future work focusing on spatiotemporal changes in the synergy between biomarkers for Aβ, tau, and neurodegeneration as it relates to progression rates is thus warranted.

## Conclusions

To conclude, our study combined data and model-driven methods to uncover the role of AT(N) biomarkers in the progression of cognitive decline along the AD continuum. The results converge to support a more complex, synergistic relationship between AT(N) biomarkers in determining this progression. Our findings further demonstrate the utility of using modeling approaches to study the complex multifaceted mechanisms that underlie disease progression in AD.

## Supplementary Information

**Additional file 1.**

## Data Availability

Data used in this study were obtained from the ADNI database (adni.loni.usc.edu), excluded in specific cases according to pre-established exclusion criterion determined by study design constraints such as MRI image resolution, availability of longitudinal data, subject’s amyloid-beta levels, etc.
