# Supplementary material for: Model-based stratification of progression along the Alzheimer disease continuum highlights the centrality of biomarker synergies
Source: Alzheimers Res Ther. 2022 Jan 24;14:16. doi: 10.1186/s13195-021-00941-1 (PMC8787915; doi:10.1186/s13195-021-00941-1)
Supplement: Supplementary file 1 — Additional file 1. [file 13195_2021_941_MOESM1_ESM.docx]

Additional Information for:

**Model-based stratification of progression along the Alzheimer disease continuum highlights the centrality of biomarker synergies**

Muhammad Usman Sadiq^1^, Kichang Kwak^1^, Eran Dayan^1,2*^ for the Alzheimer’s Disease Neuroimaging Initiative†

^1^ Biomedical Research Imaging Center (BRIC), UNC-Chapel Hill, Chapel Hill, NC 27599

^2^ Department of Radiology, UNC-Chapel Hill, Chapel Hill, NC 27599

^*^Corresponding author: Eran Dayan, Ph.D. 130 Mason Farm Road, Chapel Hill, NC 27599. Email: [eran_dayan@med.unc.edu](mailto:eran_dayan@med.unc.edu)

**Tables:**

| **Baseline attributes** | **Decline status** | |
| --- | --- | --- |
|  | MD | FD |
| MMSE | 26.09 ± 2.48 | 21.8 ± 1.52 |
| Age | 73.81 ± 7.28 | 73.20 ± 8.02 |
| Education | 15.88 ± 2.7 | 15.69 ± 2.5 |
| Gender (M/F) | 56.5/43.5 | 58.3/41.7 |

Table 1: Demographics and MMSE scores for SD/FD at baseline

| ***k*** | **Average silhouette width** |
| --- | --- |
| 2 | 0.54 |
| 3 | 0.45 |
| 4 | 0.37 |

Table 2. Silhouette analysis to determine optimal number of clusters

|  | **Tau + (CSF pTau_181_ > 21.8 pg/ml )** | **Tau -** |
| --- | --- | --- |
| **Training data** | 199 | 25 |
| **Test data** | 87 | 10 |

Table 3. Tau positivity table for subjects in the training and test sets

|  | **A** | **T** |
| --- | --- | --- |
| **Accuracy** | 0.597 | 0.567 |
| **AUC** | 0.613 | 0.509 |

Table 4. Performance of a logistic regression model used to predict cognitive phenotypes using A and T biomarkers

|  | **Accuracy** |
| --- | --- |
| N + ICV | (0.686 ± 0.021) |
| N - F | (0.677 ± 0.010) |

Table 5. Effect of adding ICV and removing FDG-PET to/from N biomarkers

**Figures:**


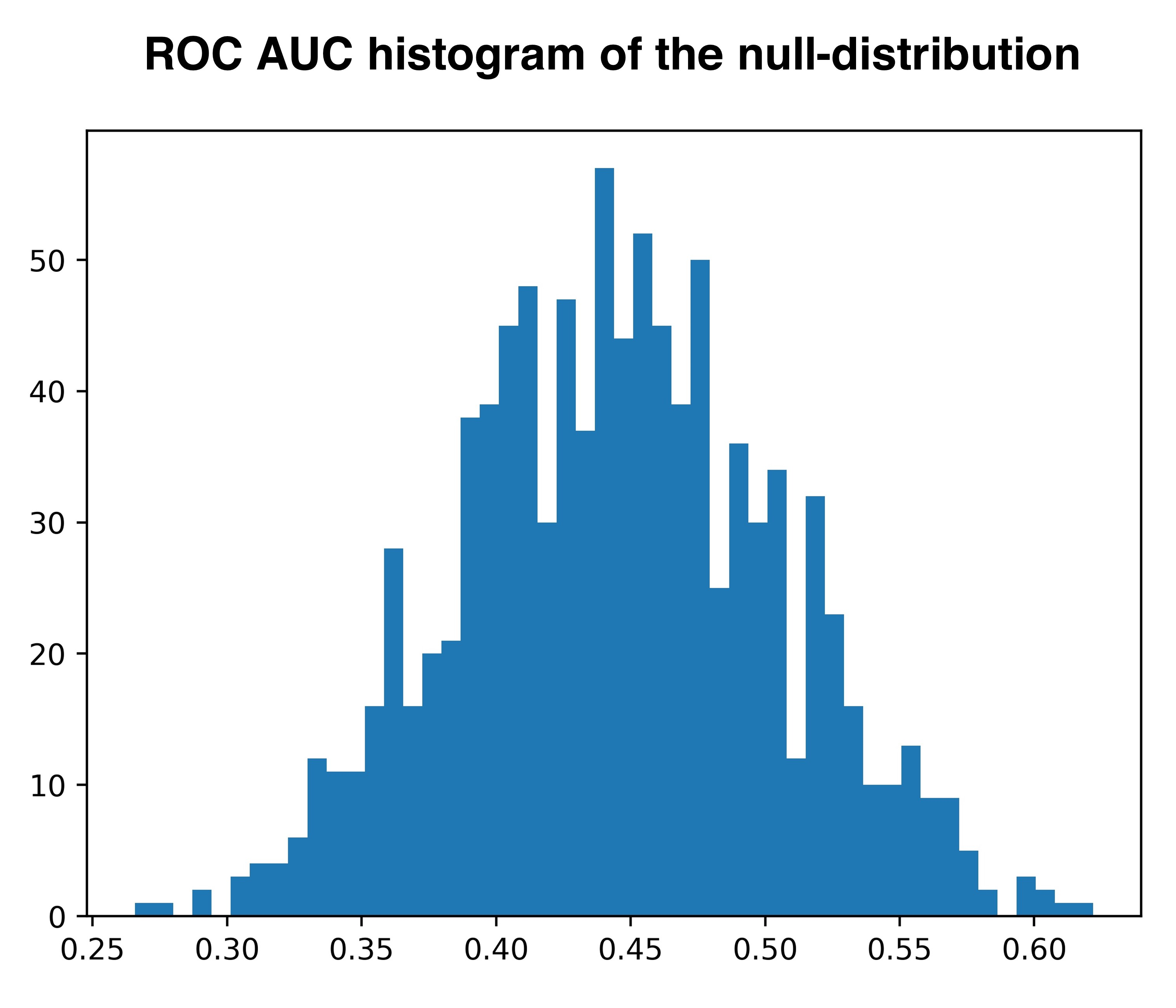


Figure S1: ROC Area-under-curve of the random classifier compared against the PENet deep learning model


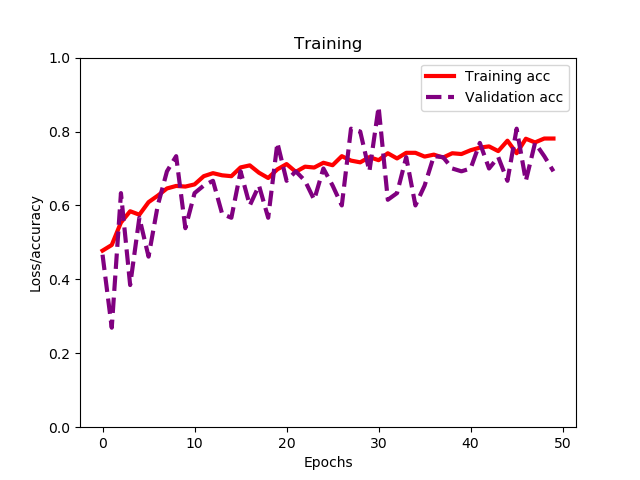


Figure S2: Training/validation accuracy curves for the PENet model under training using A/T/N features (Fold 1) do not indicate overfit


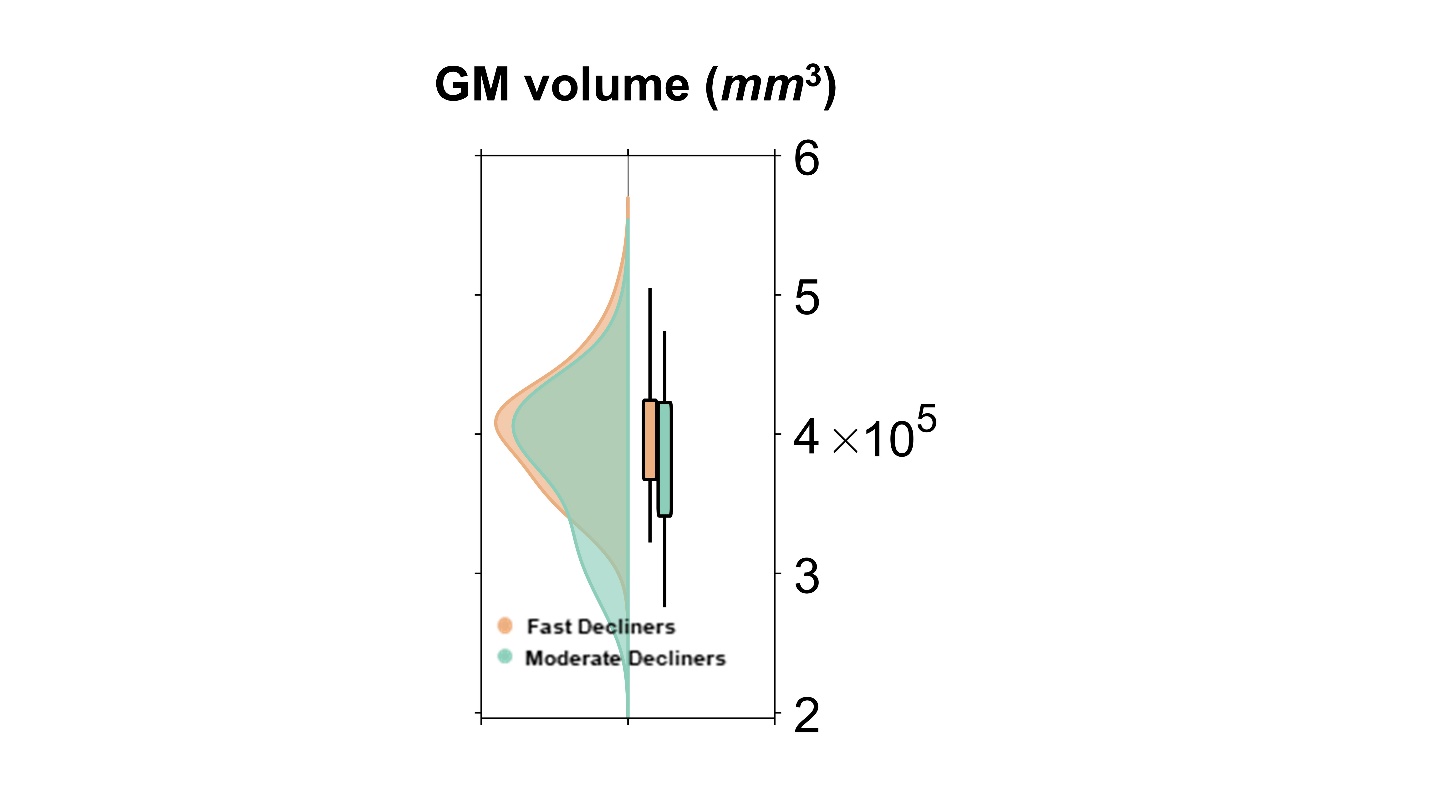


Figure S3: MD and FD phenotypes did not exhibit significant differences in total cortical volume at baseline
